# The complete chloroplast genome sequence of *Mangifera sylvatica Roxb.* (Anacardiaceae) and its phylogenetic analysis

**DOI:** 10.1080/23802359.2020.1715286

**Published:** 2020-01-21

**Authors:** Yu Zhang, Ke-Wei Ou, Guo-Di Huang, Ye-Fei Lu, Guo-Qian Yang, Xin-Hua Pang

**Affiliations:** a Guangxi Subtropical Crops Research Institute, Guangxi Academy of Agricultural Sciences, Nanning, China;; b School of Agriculture and Biology, Shanghai Jiao Tong University, Shanghai, China

**Keywords:** Chromoplast, wild mango, *Mangifera sylvatica*, phylogenetic relationship

## Abstract

In this study, we firstly reported the complete chloroplast (cp) genome sequences of the *Mangifera sylvatica* from Nanning, Guangxi province, China. The complete wild mango cp genome size is 158063 bp with a typical small single-copy region (SSC, 18340 bp), a large single-copy region (LSC, 87008 bp) and a pair of inverted repeats (IRs, 26379 bp and 26379 bp respectively). Out of 112 unique annotated genes in mango cp genome, 78 found to be protein coding, 30 to be tRNA and 4 rRNA genes. Besides, we found 51 microsatellite sequences (SSRs) in the cp genome. Sequence alignment and ML analysis of 29 full plastome data revealed *M. sylvatica* shares the closest relationship with cultivated mango (*M. indica*) and form a sister group with *Rhus chinensis* within Anacardiaceae.

Wild mango (*Mangifera sylvatica* Roxb.), which is sister to the most widely cultivated tropical fruit common mango, is an important medicinal plant belonging to the family Anacardiaceae (Baul et al. 2016). Recent studies have shown that *M. sylvatica* is of high medicinal and nutrition values as its leaves possess thrombolytic properties and fruits are rich in wild mango butter (Akhter et al. 2016). It is distributed mainly in South-east Asian countries (Baul et al. 2016). As an underutilized wild tree species, it has been threatened in Bangladesh and may go extinct due to forest degradation and climate change (Akhter et al. 2016, 2017). However, genetic resources are still scare to promote domestication and conservation of this species. Here, we assembled the cp genome of *M. sylvatica* and assessed its phylogenetic position with Illumina sequencing.

One individual plant of *M. sylvatica* was sampled from Mango Germplasm Resources Protection and Innovation Base, Guangxi Subtropical Crops Research Institute (GSCRI, 22°53′55.5″N, 108°20′33.4″E). Specimen was deposited in the Herbarium of GSCRI (HGSCRI-MS-1). Fresh leaves of the sample were immediately frozen in liquid nitrogen for DNA extraction with the Plant Genomic DNA Kit (TIANGEN, DP305). Adaptors were added to DNA the fragment and an Illumina’s Hiseq2500 sequencer was used for sequencing. After filtering out low quality data, 0.8 Gb clean data were assembled into contigs with SOAPdenovo (v 2.04) (Luo et al. 2012). Then, the assembly results were optimized according to the overlap relationships of reads with GapCloser (v1.12), and redundant segment sequences were removed to obtain the final assembly. Annotations were performed using DOGMA (Wyman et al. 2004) and adjusted according to Wang et al. (2018). Complete genome was submitted to GenBank (MK790101).

Chloroplast genome of *M. sylvatica* is 158,063 bp in length, with 37.89% GC content. The LSC is 87008 bp in length containing 82 genes, and the SSC is 18340 bp in length containing 13 genes, while two IRs are 26,379 bp containing 18 and 19 genes, respectively. 78 functional genes were annotated with 79,035 bp total length and an average gene length of 1013 bp, which is similar to the *M. indica* plastome (Azim et al. 2014; Zhao et al. 2019). Among the protein-coding genes, nine genes (rpl2, rpl16, PetB, PetD, rps16, atpF, ropC1, ndhB, and ndhA) had one intron, while rps12, ycf3 and clpP each contained two introns. tRNA genes were situated in all four regions while rRNAs are only found in IR regions. 19 LTRs and 8 DNA transposons were found scattered in the genome, while 51 SSRs were identified including mono- and di-SSR types.

To investigate the phylogenetic position of *M. sylvatica*, 29 complete chloroplast genome from 10 families were aligned by Clustalw2 (Larkin et al. 2007). ML-analysis and phylogenetic tree plotting were conducted with MEGAX (Kumar et al. 2018). *M. sylvatica* shares the closest relationship with *M. indica* and other 3 species in Anacardiaceae with 100% bootstrap support (Figure 1). Anacardiaceae forms a single clade with Burseraceae. These results were consistent with Jo’s study (2017) with only protein-coding and rRNA genes.

**Figure 1.**
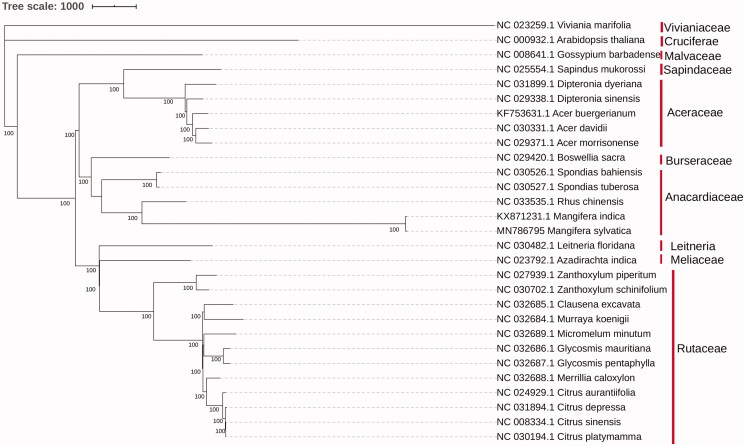
Molecular phylogenetic analysis of 29 plastomes.

The evolutionary history was inferred by using the Maximum Likelihood method based on the Jukes–Cantor model. Initial tree(s) for the heuristic search were obtained automatically by applying Neighbor-Join and BioNJ algorithms to a matrix of pairwise distances estimated using the Maximum Composite Likelihood (MCL) approach, and then selecting the topology with superior log likelihood value. Positions with less than 95% site coverage were eliminated. There was a total of 131,609 positions in the final dataset.
